# The Open Source GAITOR Suite for Rodent Gait Analysis

**DOI:** 10.1038/s41598-018-28134-1

**Published:** 2018-06-28

**Authors:** Brittany Y. Jacobs, Emily H. Lakes, Alex J. Reiter, Spencer P. Lake, Trevor R. Ham, Nic D. Leipzig, Stacy L. Porvasnik, Christine E. Schmidt, Rebecca A. Wachs, Kyle D. Allen

**Affiliations:** 1 University of Florida, J. Crayton Pruitt Family Department of Biomedical Engineering, Gainesville, 32611 USA; 2 Washington University in St. Louis, School of Engineering and Applied Science, St. Louis, 63130 USA; 3 University of Akron, Chemical and Biomolecular Engineering, Akron, 44325 USA; 4 University of Nebraska-Lincoln, Biological Systems Engineering, Lincoln, 68588 USA

## Abstract

Locomotive changes are often associated with disease or injury, and these changes can be quantified through gait analysis. Gait analysis has been applied to preclinical studies, providing quantitative behavioural assessment with a reasonable clinical analogue. However, available gait analysis technology for small animals is somewhat limited. Furthermore, technological and analytical challenges can limit the effectiveness of preclinical gait analysis. The Gait Analysis Instrumentation and Technology Optimized for Rodents (GAITOR) Suite is designed to increase the accessibility of preclinical gait analysis to researchers, facilitating hardware and software customization for broad applications. Here, the GAITOR Suite’s utility is demonstrated in 4 models: a monoiodoacetate (MIA) injection model of joint pain, a sciatic nerve injury model, an elbow joint contracture model, and a spinal cord injury model. The GAITOR Suite identified unique compensatory gait patterns in each model, demonstrating the software’s utility for detecting gait changes in rodent models of highly disparate injuries and diseases. Robust gait analysis may improve preclinical model selection, disease sequelae assessment, and evaluation of potential therapeutics. Our group has provided the GAITOR Suite as an open resource to the research community at www.GAITOR.org, aiming to promote and improve the implementation of gait analysis in preclinical rodent models.

## Introduction

Locomotive changes are often associated with disease or injury^1–3^. These compensatory gait changes may be clinically assessed as a measure of patient pain and disability. Clinical gait compensations have been well-documented^4–9^, with application in studies of neurological^10–14^ and orthopaedic diseases^15–19^, as well as sports medicine^20–23^.

Gait analysis has also been applied to preclinical studies, providing the benefit of quantitative behavioural assessment with a reasonable clinical analogue. There are several commercially available gait analysis tools, such as the CatWalk^24,25^, which have seen significant use and offer demonstrably powerful assessments. However, commercial arenas and associated software suites can be prohibitively expensive for research groups for which gait assessment is not core to their work. Furthermore, technological and analytical challenges specific to small animal studies can limit the effectiveness of preclinical gait analysis, and progress in addressing these challenges can be slowed by the proprietary nature of available commercial systems.

In this work, our Gait Analysis Instrumentation and Technology Optimized for Rodents (GAITOR) Suite is introduced as an open source gait analysis system. The GAITOR Suite includes a set of MATLAB software tools for processing gait trials from high speed videos. While our methodological approach has been previously presented with applications in osteoarthritis research^26^, new software components are presented herein for the first time, demonstrating the total system’s adaptability and application to multiple movement disorder models. The GAITOR Suite is designed to increase the accessibility of preclinical gait analysis to the research community, facilitating hardware and software customization for the needs of individual labs.

## Methods

### Hardware

**Figure 1:**
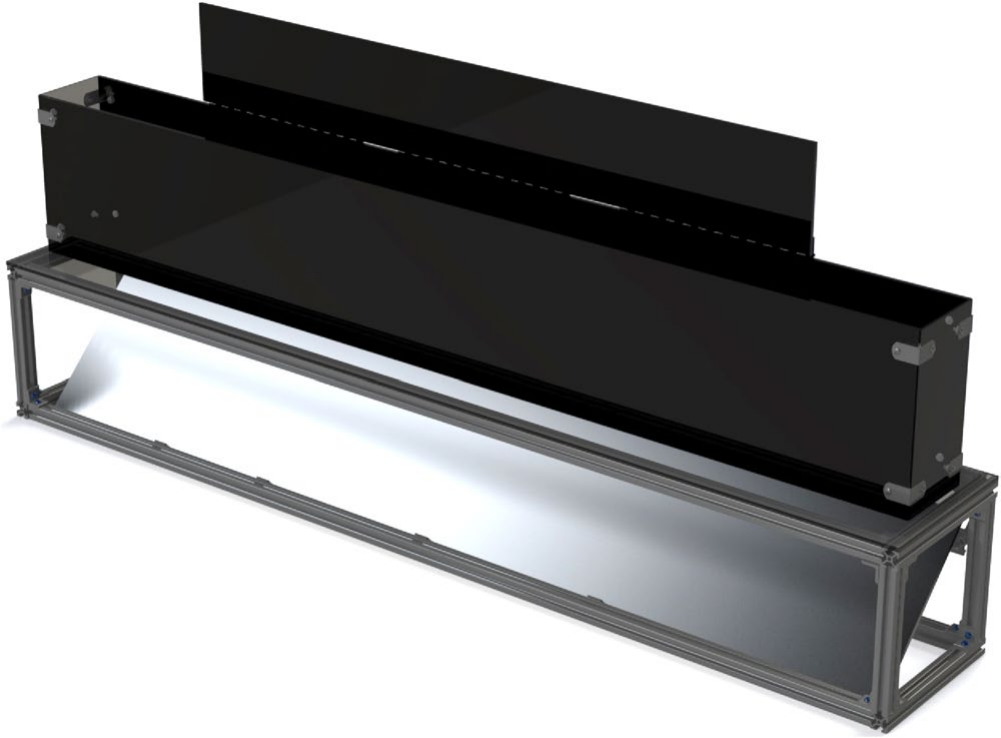
EDGAR is comprised of an 80/20 extruded aluminum base with mounted mirror and an acrylic sheet arena with a transparent floor. Build specifications and SolidWorks files are available at www.GAITOR.org.

The GAITOR Suite is designed to analyse gait trials collected with the Experimental Dynamic Gait Arena for Rodents (EDGAR), described previously^26^. EDGAR is constructed in two pieces: an acrylic arena and a base with a transparent floor and mounted mirror (Fig. 1). The arena portion of EDGAR is constructed from transparent acrylic panels, with the back wall and arena lid covered in solid coloured vinyl. To provide high contrast and limit shadows, black backgrounds are recommended for white rodents, while green backgrounds are recommended for brown or black rodents. The EDGAR base is constructed predominantly out of 80/20 and associated hardware. A mirror is housed in the base and set 45° below the arena. An acrylic sheet is placed on top of the base structure and serves as the floor beneath the arena. In our prior work, an alternate floor with instrumented force panels was designed to collect ground reaction forces during rodent gait trials. Though this work exclusively uses the non-instrumented floor, the GAITOR Suite software, described later, is compatible with either floor configuration.

The developing lab group’s arena measures 5′′ wide by 60′′ long by 10′′ tall for use with rats (providing sufficient length to capture at least four complete gait cycles). While this publication focuses on rats, EDGAR is adaptable to mice with a similar build using 3′′ wide side panels. To illuminate the arena, 15000 Lumen LED light bars are used with amber lens covers. For any EDGAR build, arena lighting should be consistent across the length of the arena and bright in both the direct lateral and reflected ventral views of the animal. Direct current lighting is recommended, as alternating current lighting has a natural flicker that can cause frame-to-frame lighting inconsistencies in high speed video. Parts used in the developing lab’s EDGAR build can be found on www.GAITOR.org, along with general suggestions and advice. Additionally, rodent gait analysis methods and recommendations are explored in greater detail in our group’s published methodological reviews^1,27^.

### Animal Models

In this work, the GAITOR Suite’s utility is demonstrated in four models: a monoiodoacetate (MIA) injection model of joint pain, a sciatic nerve injury model, an elbow joint contracture model, and a spinal cord injury model.

#### MIA Model

Twelve male Lewis rats (200–250 g) were divided into sham (n = 6) and MIA injection (n = 6) groups. Animals were anaesthetized, and the right hind limb was aseptically prepared. A 29-gauge needle was inserted behind the patella and into the femoral groove. Sham group animals were given 25 *μ*L of sterile saline, and MIA group animals were given 1 mg MIA suspended in 25 *μ*L saline. All animals received perioperative buprenorphine (0.05 mg/kg) maintained to 48 h post-surgery. Gait data were collected 4 weeks after injections. Experimental procedures were in accordance with Animal Care Services and Institutional Animal Care and Use Committee approval at the University of Florida.

#### Sciatic Nerve Injury Model

Twelve male Lewis rats (250–300 g) were divided into sham (n = 6) and allograft (n = 6) groups. Animals were anaesthetized, and the left hind limb and gluteal region of the back was aseptically prepared. A 2.5–3 cm incision was made from the femoral head to the tibiofemoral articulation. The sciatic nerve was exposed by separating the biceps femoris and gluteus muscles, and then dissected from its origin to where the sciatic nerve divides. The sham group had muscles re-approximated and the skin closed. For the allograft group, the sciatic nerve was localized and 8 mm transected, starting 4 mm from the iliofemoral band. The allograft (1 cm in length) was sutured to the proximal and distal nerve stumps using nylon sutures placed 180° apart. The muscles were reattached and skin closed. Animals received 5 mL of saline subcutaneously, and pain was managed with buprenorphine (0.03 mg/kg) for 48 h. Gait data were collected 8 weeks after surgery. Experimental procedures were in accordance with Animal Care Services and Institutional Animal Care and Use Committee approval at the University of Florida.

#### Elbow Contracture Model

Five male Long-Evans rats (320–370 g) were anaesthetized, and the left elbow was aseptically prepared. Post-traumatic joint contracture was induced, as described by Lake *et al*.^28^. Briefly, a 2 cm incision was made over the lateral elbow and the triceps retracted posteriorly such that the humerus (lateral column) was identified. The anterior capsule was cut and elevated from the humerus, and the lateral collateral ligament was transected. Additional elbow subluxation was used to increase the injury severity. The skin was then closed, and the injured limb was immobilized for 6 weeks. Animals received pre-operative enrofloxacin and pre- and post-operative carprofen. After immobilization, animals were allowed unrestricted cage activity for an additional 6 weeks. Gait data were collected 12 weeks after surgery. Experimental procedures were in accordance with Animal Care Services and Institutional Animal Care and Use Committee approval at Washington University in St. Louis.

#### Spinal Cord Injury Model

Ten female Fisher 344 rats (150–175 g) were anaesthetized, aseptically prepared, and given subcutaneous carprofen (5 mg/kg) and atropine (0.04 mg/kg). A multilevel laminectomy was performed from T7-T9 to expose the T8 spinal cord segment. A right lateral hemisection was performed using an #11 scalpel and 2 mm of cord was removed. The muscle, fascia, and skin were then closed. Carprofen and saline were given subcutaneously for 3 days. Gait data were collected 3 weeks after surgery, including gait collection on an additional 6 naive animals (litter and age-matched to injury group). Experimental procedures were in accordance with Animal Care Services and Institutional Animal Care and Use Committee approval at the University of Akron.

### Gait Data Collection

EDGAR was independently constructed at each institution. Animals were acclimated to gait arenas for 3–5 days prior to data collection. For testing, each animal independently explored an arena for 20 minutes. Animals were not prompted and walked at self-selected velocities. Videos of the animals crossing the arena were captured at 500 fps for the MIA injection and elbow joint contracture models and 250 fps for the sciatic nerve and spinal cord injury models. A video trial was saved if at least four complete gait cycles were completed at a constant velocity. At least six trials were obtained from each animal.

### Software and Data Processing

Data for the MIA and sciatic nerve injury models were processed by our group (EHL & BYJ). Data for the elbow contracture model was processed by our collaborator (AJR). Data for the spinal cord injury model was processed by our collaborator (TRH). Overarching methods for processing gait data are described below and in our prior work^26,29^. All data for these studies were processed using the GAITOR Suite software, described in detail below.

**Figure 2:**
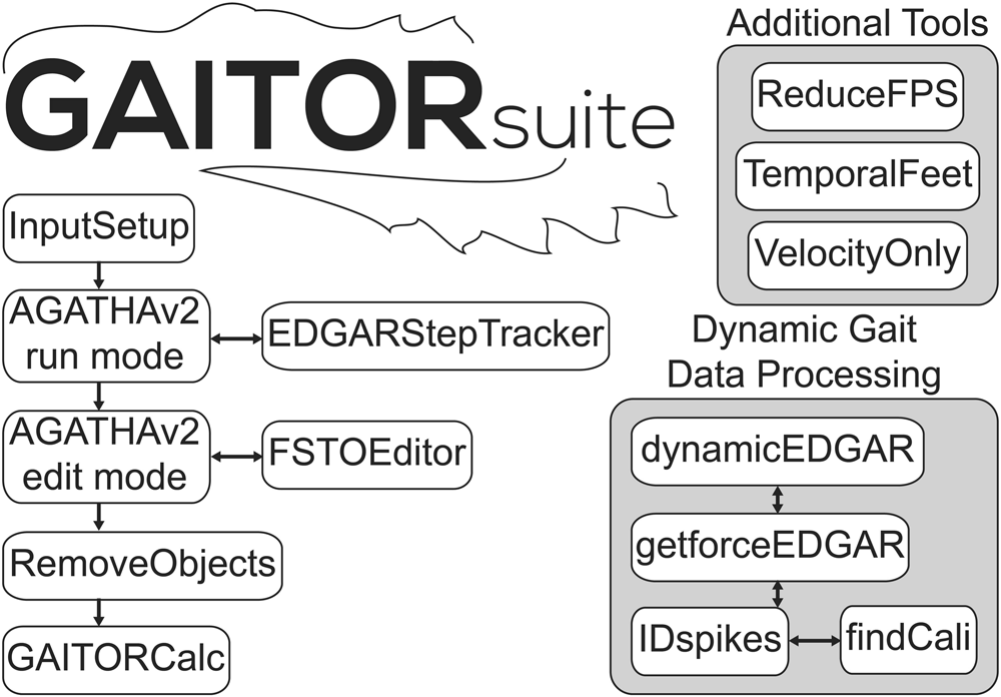
Sequence of steps taken within GAITOR Suite. Software included in the GAITOR Suite includes dynamic gait data processing scripts and additional tools.

**Figure 3:**
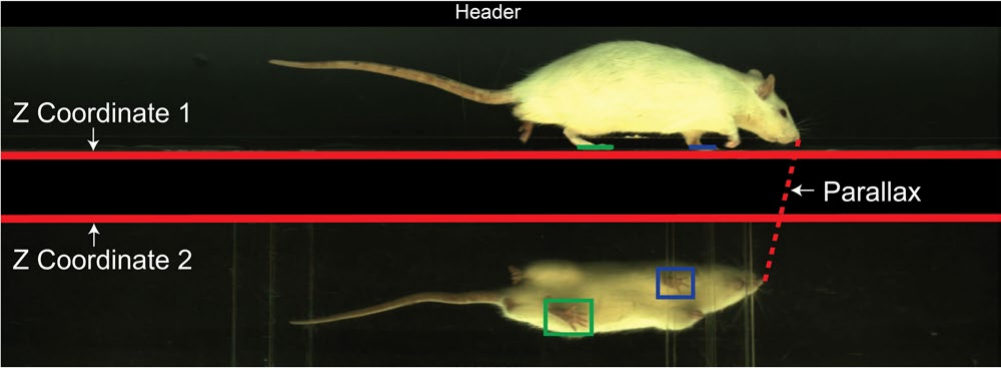
Each frame of a video collected with EDGAR is broken down by AGATHAv2 to track the location of paws (blue and green boxes) and the timing of foot-strike, stance, and toe-off (blue and green lines tracked frame by frame). AGATHAv2 can identify these events accurately after users set input parameters, such as the location of the floor (Z Coordinate 1), the demarcation beginning the ventral arena view (Z Coordinate 2), parallax adjustment, and whether a video header overlay should be cut from each frame. These parameters are set using the GAITOR Suite’s input script.

GAITOR Suite software includes, among other tools, a simplified set-up script for users without coding experience and a video processing software called AGATHAv2 (Fig. 2). AGATHAv2 is a ground-up rebuild of our group’s previously published Automated Gait Analysis Through Hues and Areas (AGATHA)^29^. At its core, AGATHAv2 still served to identify a rodent’s paws in a gait trial video and track them both spatially and temporally (Fig. 3). AGATHAv2 was written to provide AGATHA functionality for a broad range of lighting conditions, improve filtering algorithms for rodent identification and gait trial events, and provide adaptability for different rodent species and strains. AGATHAv2 was also written to interface with GAITOR Suite’s tools and input script, created to make batch processing large data sets customizable and more user friendly.

#### Batch Set-up

To begin, users ran the “InputSetup” script included with the GAITOR Suite. The script is divided into several segments, including main parameters, video adjustments, and troubleshooting and display options. Main parameters should be set for each batch, as video capture conditions may be variable between research groups or even collection sessions within individual experiments. Factors like distance of camera from arena, camera type, and arena levelling all impact video capture variability. To minimize the effects of recording session variability on video processing quality, GAITOR Suite’s set-up script allows users to refine processing for each batch.

To further control these factors, gait data for each model were processed in batches that represented a single day of data collection, ensuring consistent lighting and arena conditions. Variables set in the input script include the location of the arena floor (Z Coordinate 1), the top of the ventral arena view (Z coordinate 2), and a parallax correction factor (used to adjust for parallax effects which scale to the left and right of the image centre line (Fig. 3). These batch parameters help AGATHAv2 accurately determine when and where a paw is in contact with the floor. Descriptions of each variable and all default values are provided in Tables 1 and 2. Furthermore, comments explaining each variable and guiding users through appropriately defining variables for a given batch are provided in the script. Typically, a new user will only be required to make batch-specific adjustments within the main parameters section of the script. Nonetheless, documentation for more advanced users and unique cases are addressed at www.GAITOR.org.

**Table 1:** Core GAITOR Suite software used to process spatiotemporal gait data.

GAITOR Suite Core Software	
Script/Function	Use
InputSetup	Set batch parameters and activate/deactivate AGATHAv2 features.
AGATHAv2	Process video trials to collect raw spatial and temporal event data.
RemoveObjects	Trim AGATHAv2 trial data down to constant velocity segments.
GAITORCalc	Calculate spatiotemporal gait parameters from trial event data.

**Table 2:** Video Adjustment and EDGAR Options parameters are set by the user within InputSetup.

Setup Parameters (InputSetup)	
Variable	Definition & Defaults
Batch	Identifier for video batch; input files save as “Input_Batch”
XParameters	Defaults are [1 0]; sets the frame region of interest to assess
FrameParameters	Defaults are [1 0]; sets the starting and ending frame of the video
VidFPS	Input the video frame rate in frames per second
ZCoordinates	Set the coordinate for the floor and top of the ventral view (y-coordinate in a MATLAB displayed image)
RatSize	Set the lower threshold for number of pixels needed to qualify as the rat object in both the lateral and ventral views
Parallax	Accounts for distortion caused by the mirror; typical parallax values are around 10% (0.1)
Header	Default is ‘N’; set to ‘Y’ if videos have a header (camera specific) that should be removed
TailCutter	Default is ‘N’; set to ‘Y’ if TailCutter should be disabled and the tail should not be filtered from the animal image
EDGAR	Default is ‘N’; set to ‘Y’ if gait collection was conducted using a dynamic gait capable version o EDGAR
PlateBounds	Only change if EDGAR is set to ‘Y’; sets the x location (min and max) for the boundaries of each instrumented force panel
Display	Default is ‘Quite’; sets which images to display (do not change unless troubleshooting as it will slow down processing time)
OutputFigs	Default is ‘N’; change to ‘Y’ if output images should be displayed during processing (this setting will not affect image saving)
VideoPreload	Default is ‘N’; can be changed to ‘Y’ for troubleshooting to avoid reloading a video repeatedly

Users may choose to activate features, such as video header trimming, by changing the value of the respective variable (i.e. “Header”) as described in the code comments.

**Figure 4:**
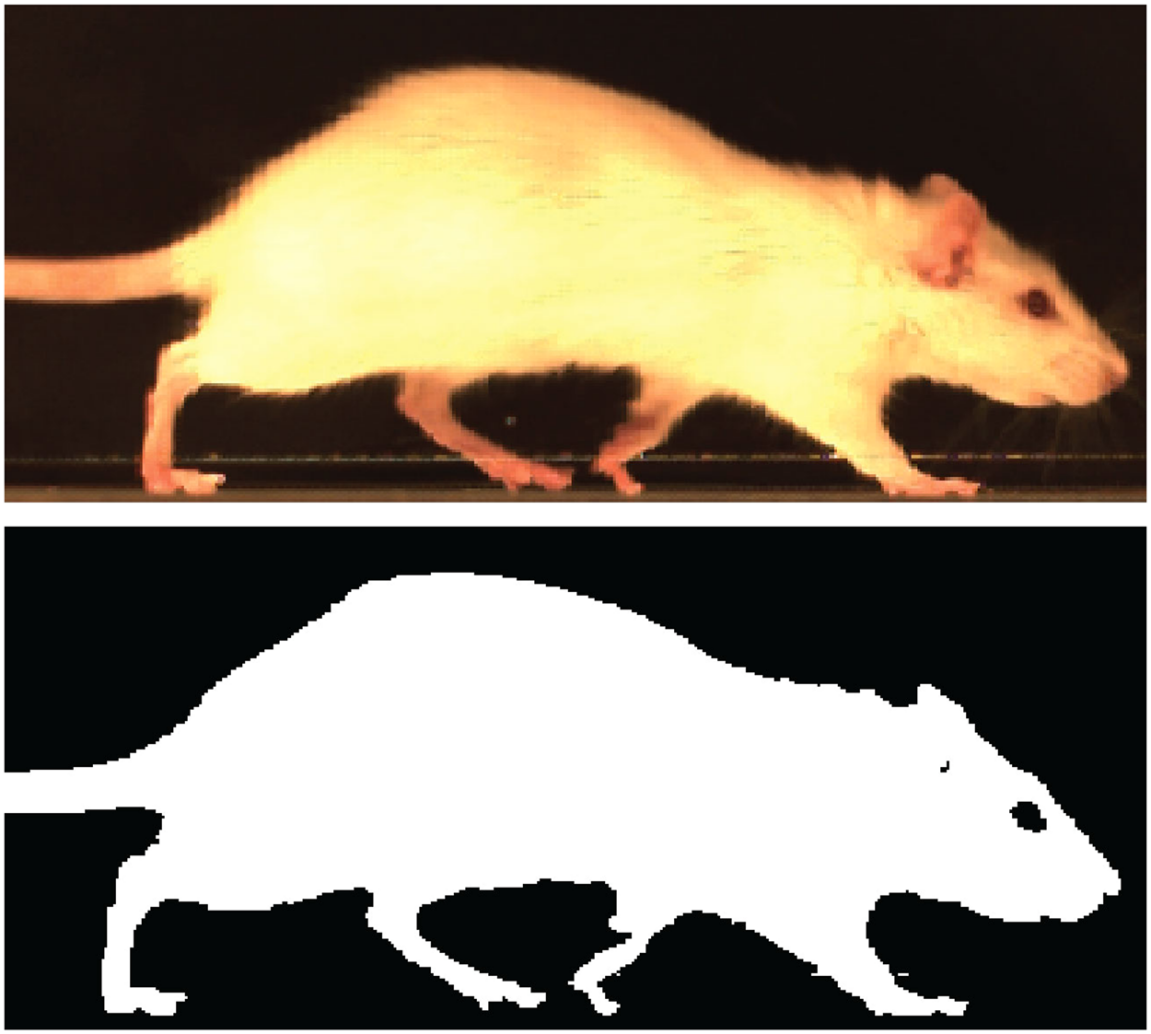
A lateral view of the rat is used to create the rat filter that AGATHAv2 uses to isolate the animal.

After running the input script, MATLAB’s colorThresholder was used to create two filters. One filter is used to isolate the rat from the lateral and ventral views (Fig. 4), while the second filter isolates the paws from the ventral view. To be clear, these filters are used to mask and create binarized images isolating desired objects (rat or pawprints). MATLAB’s colorThresholder allows users to create RGB, HSV, YCbCR, and/or L*a*b colour space filters. Details on creating multi-colour space filters are included in GAITOR Suite code documentation. As colorThresholder is a MATLAB-provided application, documentation for its basic use can be found through MathWorks’ online documentation. Basic instructions on filter creation are also provided in the help files bundled with the GAITOR Suite.

#### Automated Digitization

Set-up script outputs, filters, and the associated batch of videos were passed to AGATHAv2. Digitizing the rodent gait sequence requires identifying the frame and position of foot-strike and toe-off events (Fig. 4). AGATHAv2 automates this process, isolating and identifying the rat (lateral view) and its paws (ventral view) in each frame.

**Figure 5:**
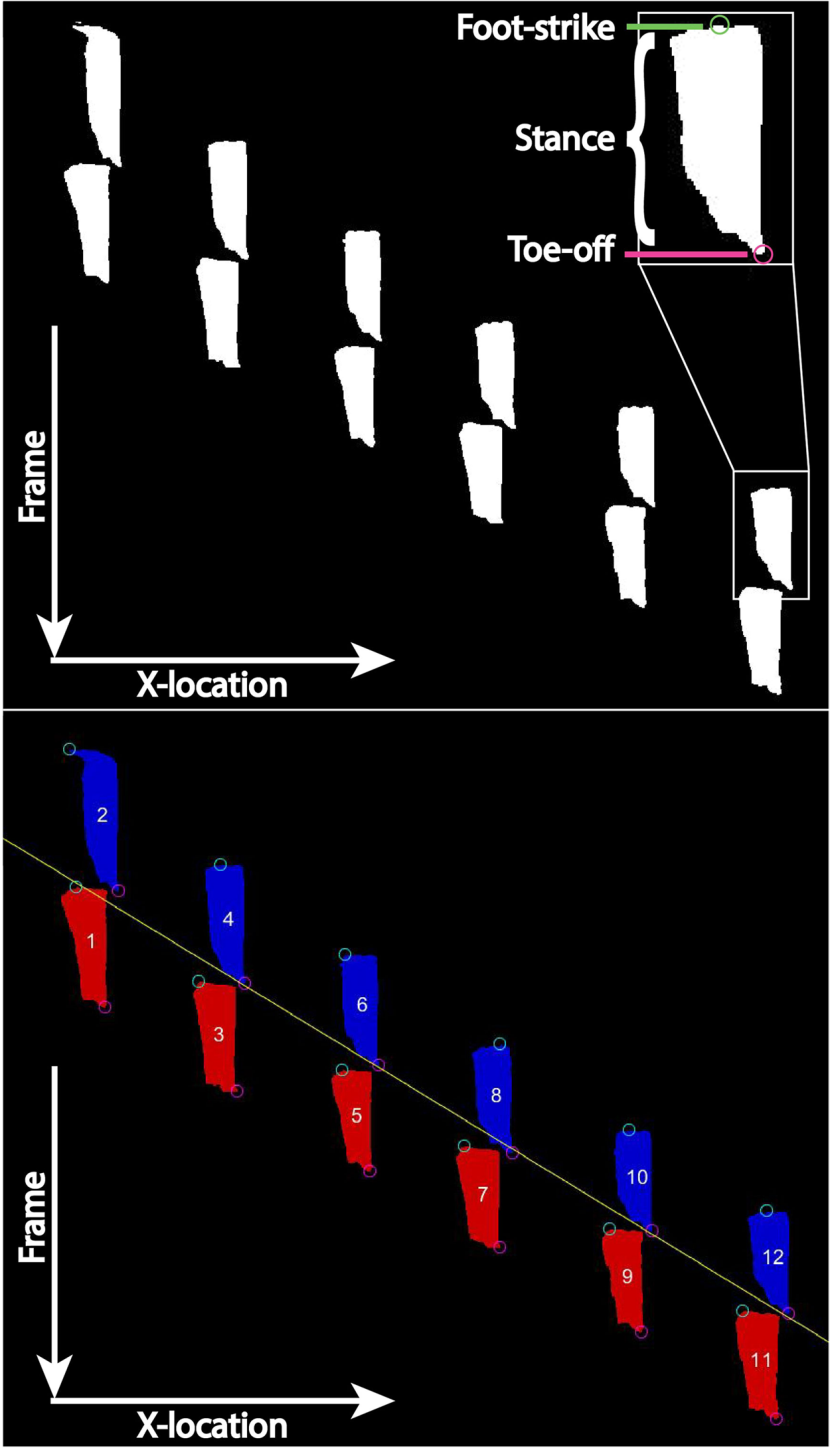
Foot-strike (green circle) and toe-off (pink circle) can be identified as the uppermost and lowermost pixels in each white footstep object, respectively. Footstep objects are additionally identified as hind (red) or fore (blue) paws.

In the lateral view, AGATHAv2 filters each video frame such that the animal appears as a white silhouette on a black background. The bottom row of white pixels marks the location of the rat’s foot on the arena floor (Fig. 4). By sampling the bottom set of rows from each frame, a foot-strike and toe-off (FSTO) image can be generated (Fig. 5), where paws in contact with the floor will appear as white segments on an otherwise black line. In this FSTO image, the “floor rows” of pixels from each frame are stacked vertically to create an image where the y-axis indicates time in frames and the x-axis indicates position in the arena. The white portions of stacked pixel rows form temporal footstep objects, where the uppermost pixel of an object is foot-strike, and the lowermost pixel of the object is toe-off (Fig. 5). From these objects, the following temporal gait parameters can be calculated: stance times, stride times, imbalance, and temporal symmetries^1,27^.

**Figure 6:**
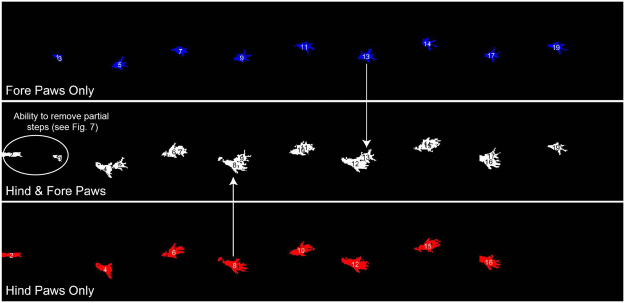
Paw print objects for the trial are displayed on a single figure. From this image, spatial measures can be taken based on the centroid locations of the paw print objects. Paw print objects are additionally identified as hind (red) or fore (blue) paws.

In the ventral view, AGATHAv2 identifies paw locations, which can be used to calculate spatial measures. Paw prints can be isolated in the ventral view during the frame sequences corresponding to each step. The resulting paw print image (Fig. 6) is reminiscent of the footprint test^30^. Spatial measures (step widths, stride length, and spatial symmetries) can then be calculated based upon the location of each paw print in the sequence.

Finally, AGATHAv2 tracks rodent velocity via the animal’s centroid (both lateral and ventral views), as well as the animal’s nose. Ventral view centroid tracking provides the most accurate measure of animal velocity and is used for all calculations in this work. Lateral view centroid tracking and nose tracking calculations of velocity are maintained as secondary checks for velocity tracking and filter quality.

To be clear, with the exception of calling the AGATHAv2 function, users are not required to interact with the program during batch processing. AGATHAv2 is fully autonomous in its ‘Run’ mode configuration, and the aforementioned processes are handled without user input.

#### Spatiotemporal Parameter Calculation

**Figure 7:**
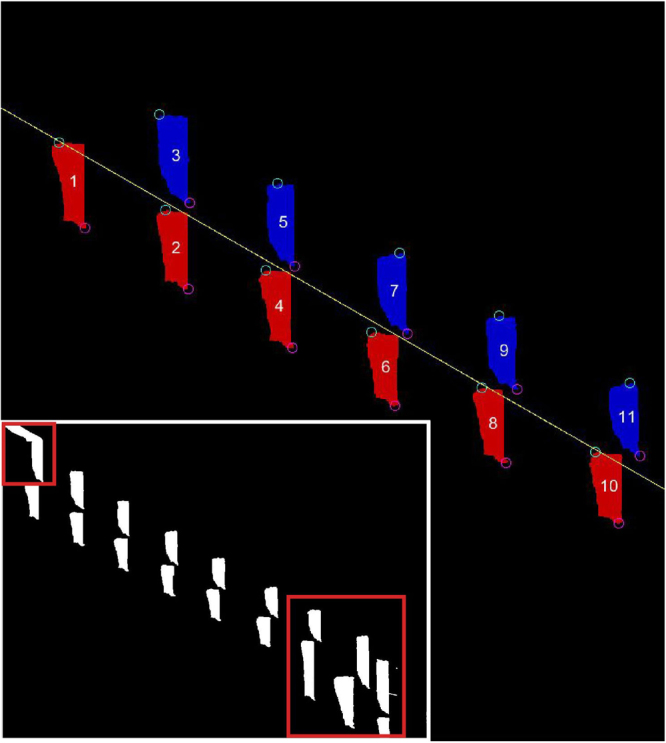
Using GAITOR Suite editing tools, objects with artefacts (small red box) or segments of trials with changing velocity (large red box) can be removed from the raw FSTO image (inset). The edited FSTO data (pictured in colour) can be passed to the GAITOR Suite calculator.

After running a batch of videos through AGATHAv2, the GAITOR Suite’s editing tools (AGATHAv2 ‘Edit’ mode and RemoveObjects) can be used to remove artefacts and unacceptable trial segments, such as where an animal paused or changed velocity (Fig. 7). This allows users to focus on specific segments of a video without reprocessing or discarding the trial. For most users, setting AGATHAv2 to ‘Edit’ mode is sufficient to edit trials. AGATHAv2’s ‘Edit’ mode provides a series of user prompts, guiding users through the process of editing trials. In addition, help documentation is provided in the GAITOR Suite bundle.

After finalizing a dataset from AGATHAv2, the GAITOR Suite calculator (GAITORCalc) returns spatiotemporal gait parameters for both fore and hind paws: stride length, step width, spatial symmetry, duty factor, imbalance, and temporal symmetry.

The GAITOR Suite’s calculator was written with user modification in mind. The calculator accepts output data from AGATHAv2, which characterizes the gait sequence as a series of objects (described by the FSTO and pawprint images). As such, users have access to spatially and temporally sorted matrices of their trial data, including x and y coordinates describing each step’s centroid, foot strike, toe off, and identifying data (left vs. right, fore vs. hind, and object number). Thus, new sections using these data can be easily appended to the calculator script without altering the core, imaging processing functions contained in AGATHAv2.

### Statistics

Data for the MIA injection model, sciatic nerve transection model, and joint contracture model were compared to a historical database of spatiotemporal data for gender matched naive rats, with residuals calculated to account for the effects of velocity and body weight covariates (as described in our methodologic review)^27^. This historical database includes gait data from naive rats dating back to 2010 and has been shown to contain consistent measures of naive gait patterns, regardless of the data analysis method^31^. Data for the spinal cord injury model were compared to data from 6 naive gender and age matched rats, with residuals calculated to account for the effects of velocity. T-tests were run to compare experimental data against expected values (0.0 for residuals or 0.5 for symmetry measures). T-tests were also used to compare experimental groups to sham groups, when present in the experimental design. For all statistics, p-values less than 0.05 are considered statistically significant. There were no controls for potential false discoveries in the analysis of these studies, as these were small-scale pilot studies assessed to demonstrate the utility of the GAITOR Suite.

### Data availability

The datasets generated during and/or analysed during the current study are available from the corresponding author on reasonable request.

## Results

Results for each model are summarized below, with figures and discussion relating to the most significantly changed gait parameters in each model. All animal data in this manuscript is being presented for the first time, with the exception of MIA model data. The MIA model data discussed in this work is a subset of data presented in a recently published study by our group^31^. It should be noted that these data were analysed independently of the analysis presented in the Lakes *et al*. publication^31^.

### MIA Injection Model

**Figure 8:**
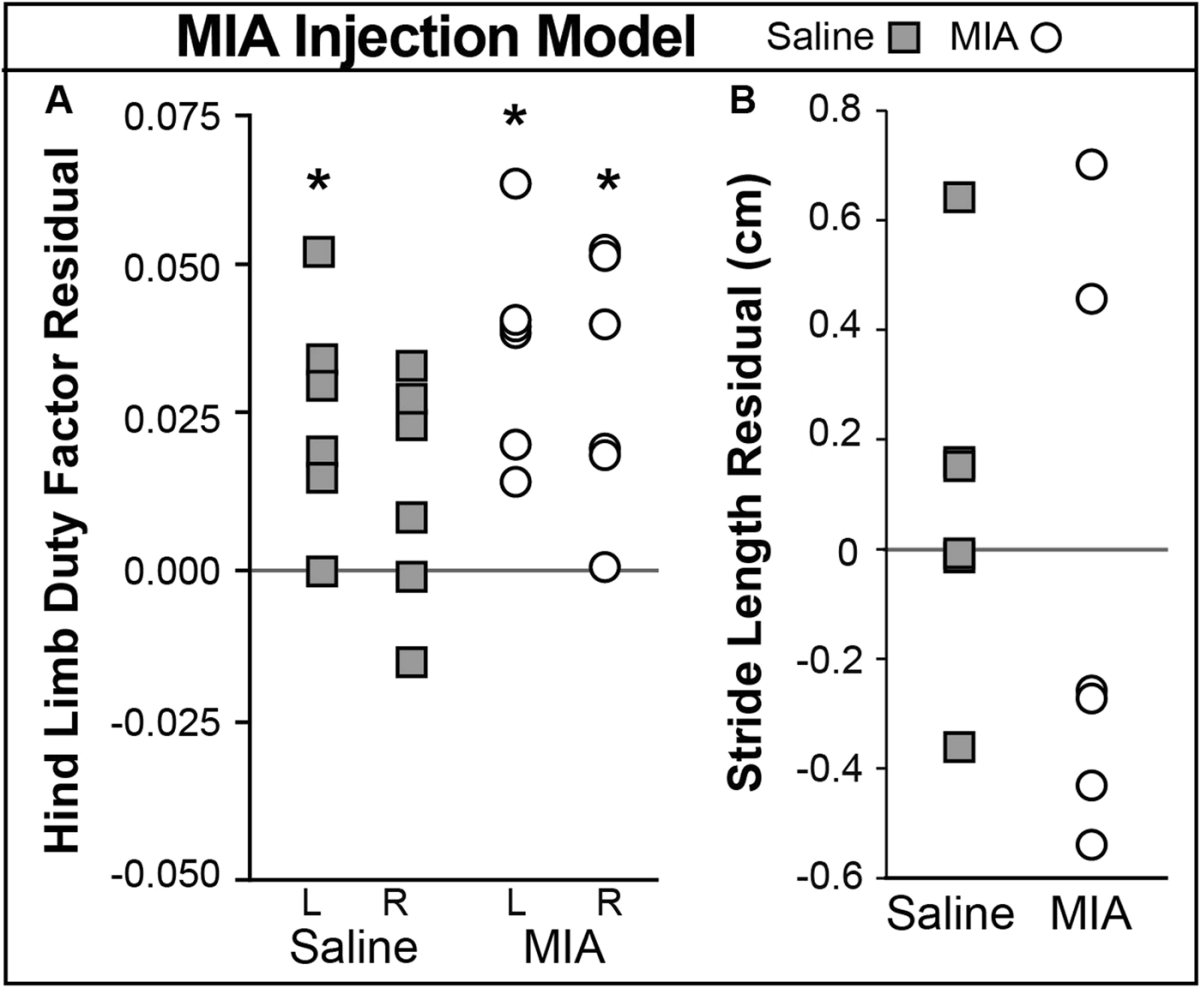
MIA Injection Model Results. Injections (MIA or saline) were delivered to the animals’ right knees. Asterisks indicate significant difference (p < 0.05) from expected values, which are indicated by solid, horizontal lines. (**A**) Left hind limb duty factor was significantly elevated for the MIA group, and also trended higher for the right limb. (**B**) No significant changes were seen in stride length, although MIA animals may tend to have shorter strides.

MIA injected animals presented higher than expected duty factors in both hind limbs (Fig. 8, left p = 0.004, right p = 0.017). Since both left and right hind limb duty factors were elevated, this low dose MIA model may cause a shuffling gait at later time points, where both hind limb duty factors are elevated. Here, the animal remains balanced as they spend more time in stance on each foot. Surprisingly, saline injected animals also had a significantly higher left hind limb duty factor compared to expected values (Fig. 8, p = 0.020).

### Sciatic Nerve Injury Model

**Figure 9:**
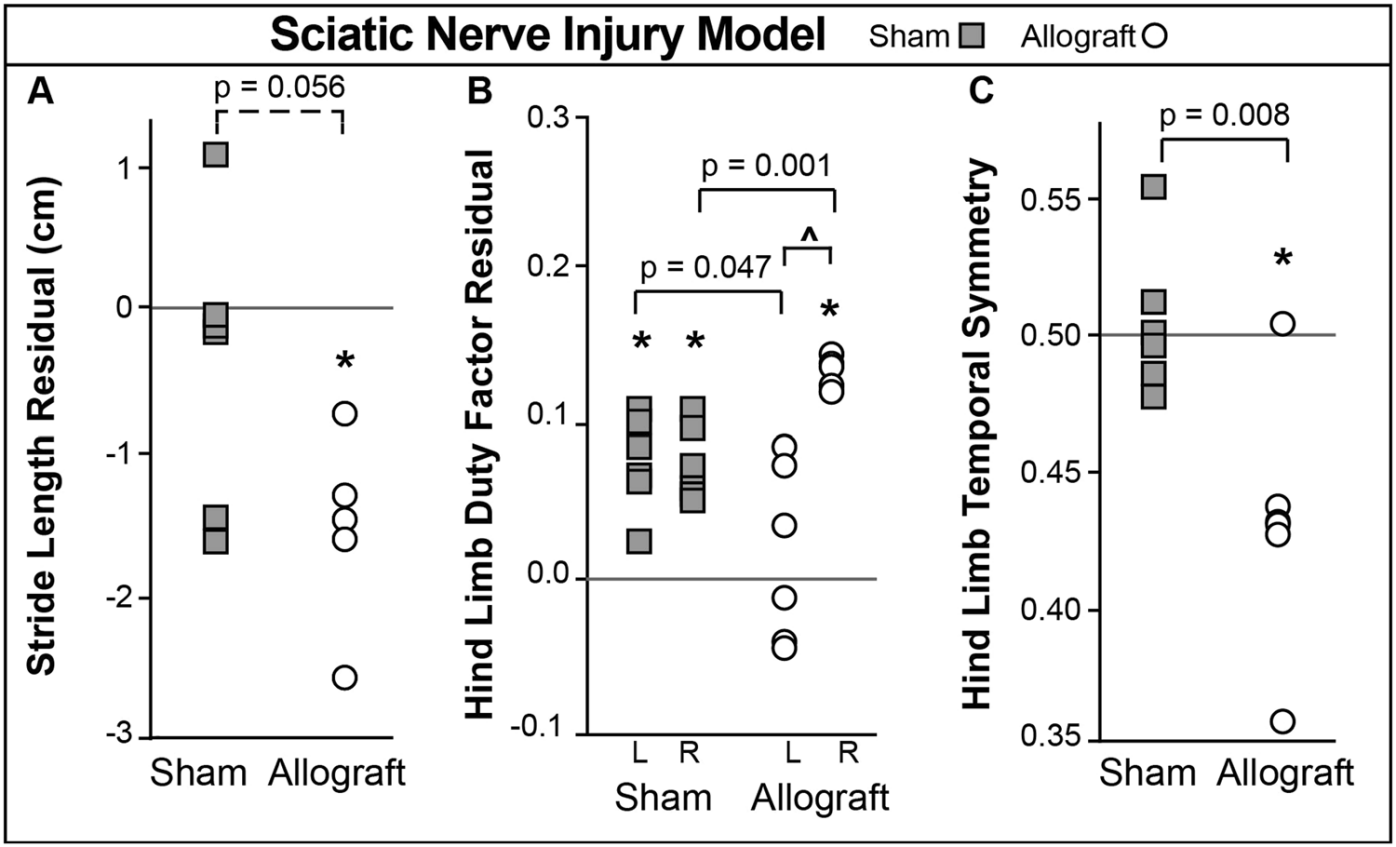
Sciatic Nerve Transection Model Results. Surgical procedures were performed on the animals’ left hind limb. Asterisks indicate significant difference (p < 0.05) from expected values, which are indicated by solid, horizontal lines. (**A**) Stride lengths were shorter in allograft animals. (**B**) Hind limb duty factors were elevated in sham group animals, while allograft group animals had higher than expected duty factors in the right limb alone. Allograft animals were also imbalanced (indicated by ^). (**C**) Allograft animals were temporally asymmetric.

Sham surgery animals presented higher than expected duty factors in both hind limbs (Fig. 9, left p = 0.002, right p < 0.001), indicating a shuffling gait. These animals did not show increased stride lengths, though allograft group animals had shorter stride lengths (Fig. 9, p = 0.007). Allograft animals were also imbalanced (Fig. 9, p = 0.003), spending more time on their right limb (Fig. 9, p < 0.001). Compared to expected values and compared to sham animals, allograft animals were temporally asymmetric, where right foot strikes occurred sooner than expected (Fig. 9, p = 0.014 and p = 0.008 respectively). These data indicate allograft animals exhibited an antalgic gait, or limp, favouring protection of the left hind limb (surgically affected side).

### Elbow Contracture Model

**Figure 10:**
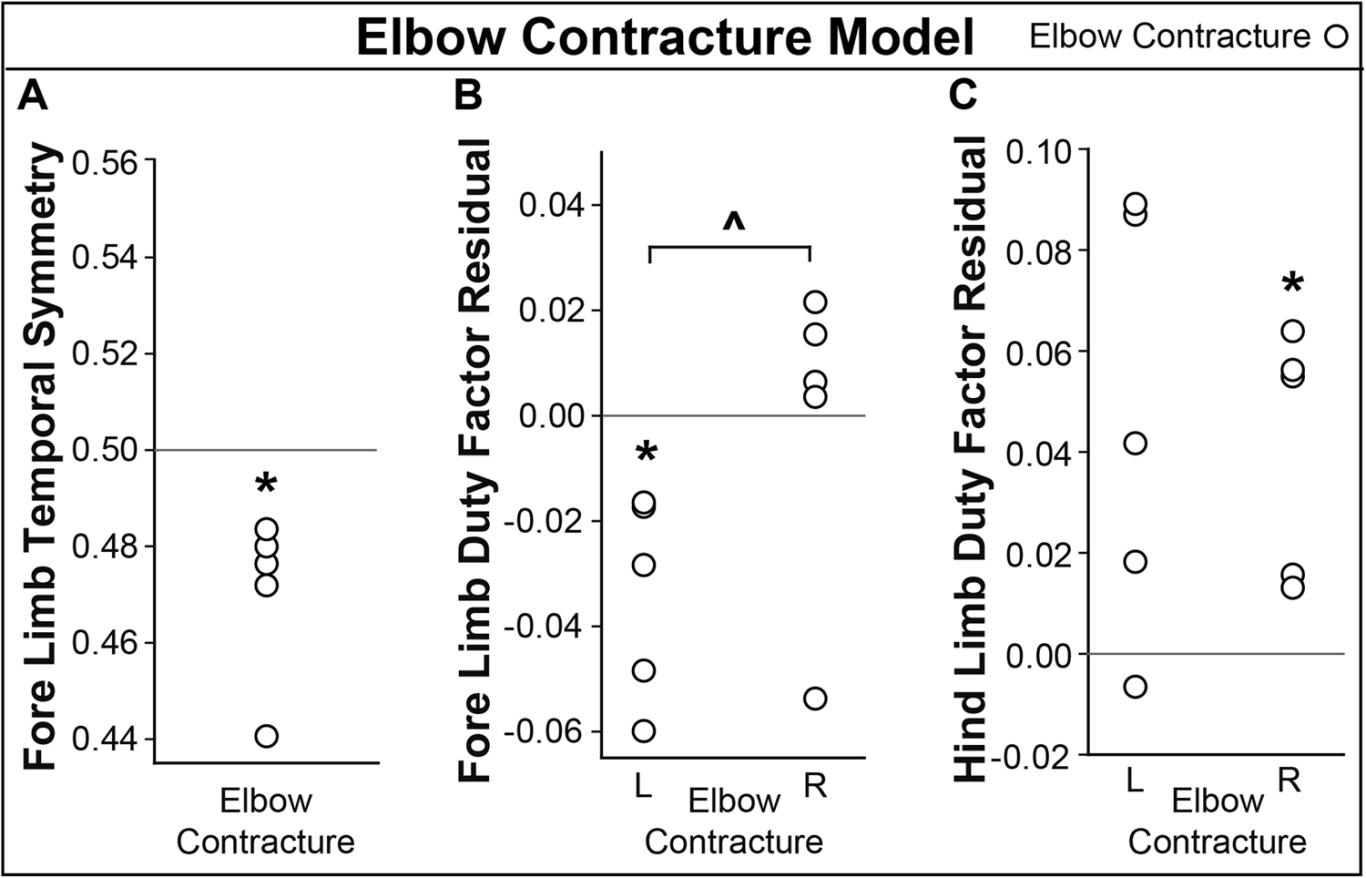
Elbow Contracture Model Results. Surgical procedures were performed on the animals’ left fore limbs. Asterisks indicate significant difference (p < 0.05) from expected values, which are indicated by solid, horizontal lines. (**A**) Elbow contracture animals were temporally asymmetric compared to expected values. (**B**) Fore limb duty factor indicated less time was spent on the left (injured) fore limb than expected. The fore limbs were also imbalanced (indicated by ^). (**C**) Hind limb duty factor showed more time spent on the right hind limb than expected; a trend (non-significant) also seen in the right fore limb.

Injured animals were temporally asymmetric compared to expected values (Fig. 10, p = 0.018), with fore limb temporal symmetry below 0.5. This indicates the right fore foot strike occurred sooner than expected. In the injury group animals, left fore limb duty factor was lower than expected (Fig. 10, p = 0.017), indicating less time spent in stance on the injured fore limb than a healthy animal. Corresponding to the decrease in left fore limb duty factor, four of five injured rats had increased fore limb duty factors (Fig. 10, non-significant). While the increase in right fore limb duty factor was non-significant, injured animals were imbalanced in the fore limbs (Fig. 10, p = 0.019). In the hind limbs, duty factors tended to raise on both the left and right limbs, with increases on the right limb being statistically significant (Fig. 10, p = 0.020) These data indicate an antalgic gait, where the limp is seen in the animal’s fore limbs.

### Spinal Cord Injury Model

**Figure 11:**
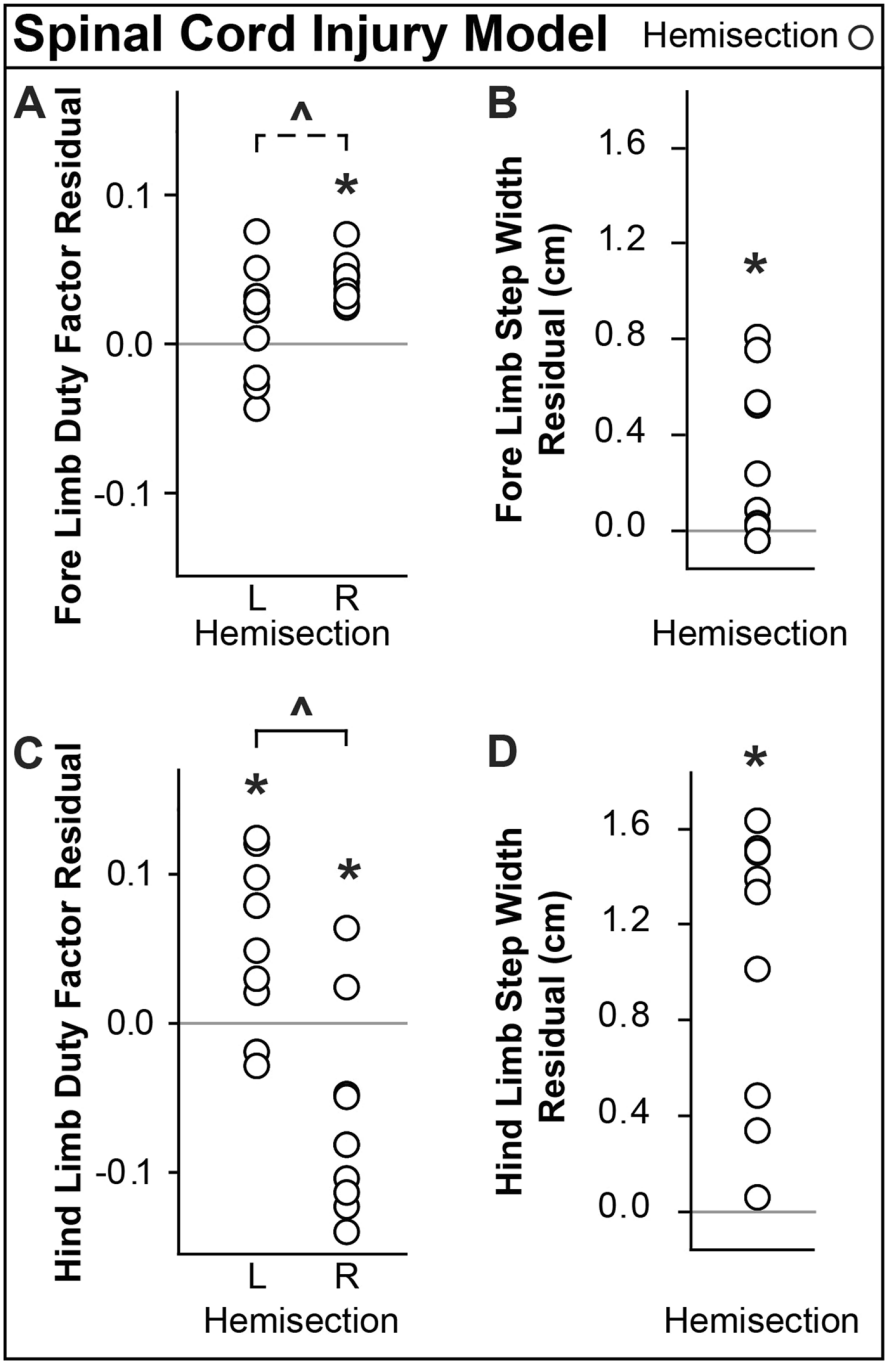
Spinal Cord Injury Model Results. A right lateral hemisection of the T8 spinal cord segment was performed on injury group animals. Asterisks indicate significant difference (p < 0.05) from expected values, which are indicated by solid, horizontal lines. (**A**) Spinal cord injury animals had higher right fore limb duty factors than expected and tended to be imbalanced relative to the left fore limb (indicated by the dashed line and ^). (**B**) Fore limb step width of injured animals was higher than expected. (**C**) Hind limb duty factor showed more time spent on the left hind limb and less time spent on the right hind limb than expected. Left and right hind limbs are also imbalanced (indicated by ^). (**D**) Hind limb step width of injured animals was higher than expected.

Hemisection animals tended to be imbalanced between left and right fore limbs compared to age-matched naive animals (Fig. 11, p = 0.054). Injured animals also spent significantly more time on their right fore limb (Fig. 11, p < 0.001) and left hind limb (Fig. 11, p = 0.009) than naive animals. Since the right fore limb and left hind limb are typically in stance at the same time, these results indicate that modifications to both fore limb and hind limb duty factors work together to offload the affected right hind limb (significantly lower duty factor than naive animals, Fig. 11, p = 0.013). Additionally, injured animals had increased step widths for both fore and hind limbs relative to expected values (fore p = 0.026, hind p < 0.001). These data indicate a severe antalgic gait developed in both the fore and hind limbs.

## Discussion

In this work, the GAITOR Suite was used to identify compensatory gait patterns in models of joint pain, sciatic nerve transection, elbow contracture, and spinal cord injury. With our system, the development of unique gait compensations were identified in each model. Furthermore, the GAITOR Suite was able to identify and quantify the changes in each model, despite different presentations of injury severity to the naked eye. As such, these data demonstrate the utility of the GAITOR Suite to detect relevant gait changes with clinical analogues in rodent models for highly disparate injuries and diseases.

With the exception of the spinal cord injury model, it is worth noting the velocity and body weight corrected data were calculated using a naive male Lewis rat database dating back to 2010. This was due to the use of female animals in the spinal cord injury model; thus, data in this model was velocity corrected using a 6 animal gender-matched naive cohort. Furthermore, we acknowledge that strain differences may impact rodent gait (the elbow contracture model utilized Long-Evans rats while our naive database is comprised of Lewis rats); however, a historical database of naive Long-Evans gait was not available. Moving forward, our group is improving our existing naive database by including both male and female trials and varying animal strains.

### MIA Model

The MIA model resulted in a shuffling gait compensation, where animals moved cautiously and spent longer than expected on both hind limbs. Shuffling gaits can often arise in response to proprioceptive deficits, instability, or pain. Interestingly, saline injected animals also exhibited slight gait modification. Saline animals walked with gaits where duty factor was higher in the left limb (saline injections were delivered to the right knee only), indicating animals spent more time on their left limb. This gait adaptation may result from the joint injections, where the establishment of a maladaptive gait may not necessarily resolve over time. Animals may develop tentative gaits due to the stress from the procedure, altered proprioception from capsular stretch, or dysesthesia from the injection. Thus, while saline was not anticipated to cause deviations from naive gaits, adaptations in response to the injection procedure may have resulted in the establishment of a gait change that persisted weeks after the initial “injury.” These findings would benefit from the added insight of longitudinal gait testing and additional measures of sensitivity, such as von Frey filament testing, in future studies.

While different from the anticipated result, these data help demonstrate the sensitivity of the GAITOR Suite platform, where subtle changes were seen in a low dose MIA model despite relatively minor joint damage (per histology, not shown). Not only were subtle changes found after 1 mg MIA injection, but gait changes were also detected in saline injected animals. This finding was not anticipated, but highlights the power of the GAITOR Suite and the need to compare experimental groups to multiple controls.

### Sciatic Nerve Transection Model

In the sciatic nerve injury model, both antalgic and shuffling gaits developed. Here, allograft group animals developed antalgic gaits while sham surgery animals developed a shuffling gait. As mentioned previously, these compensations may develop for a number of reasons. Both acute and chronic pain may lead to the development of either antalgic or shuffling gait compensations, and the unique compensations observed between these groups may be related to severity of pain and disability or associated with additional proprioceptive or mechanical dysfunctions. Cautious, shuffling gaits are often associated with proprioceptive or balance deficits. Thus, identification of the shuffling gait in animals who did not receive an allograft intervention may indicate the persistence of sensorimotor deficits due to surgery. The antalgic gait seen in the allograft group animals was consistent with left-sided injury, where animals used shorter strides and spent longer on their uninjured right limb. These data would suggest the allograft group animals did not yet experience full resolution of symptoms, or developed a persistent gait abnormality prior to symptom resolution.

**Figure 12:**
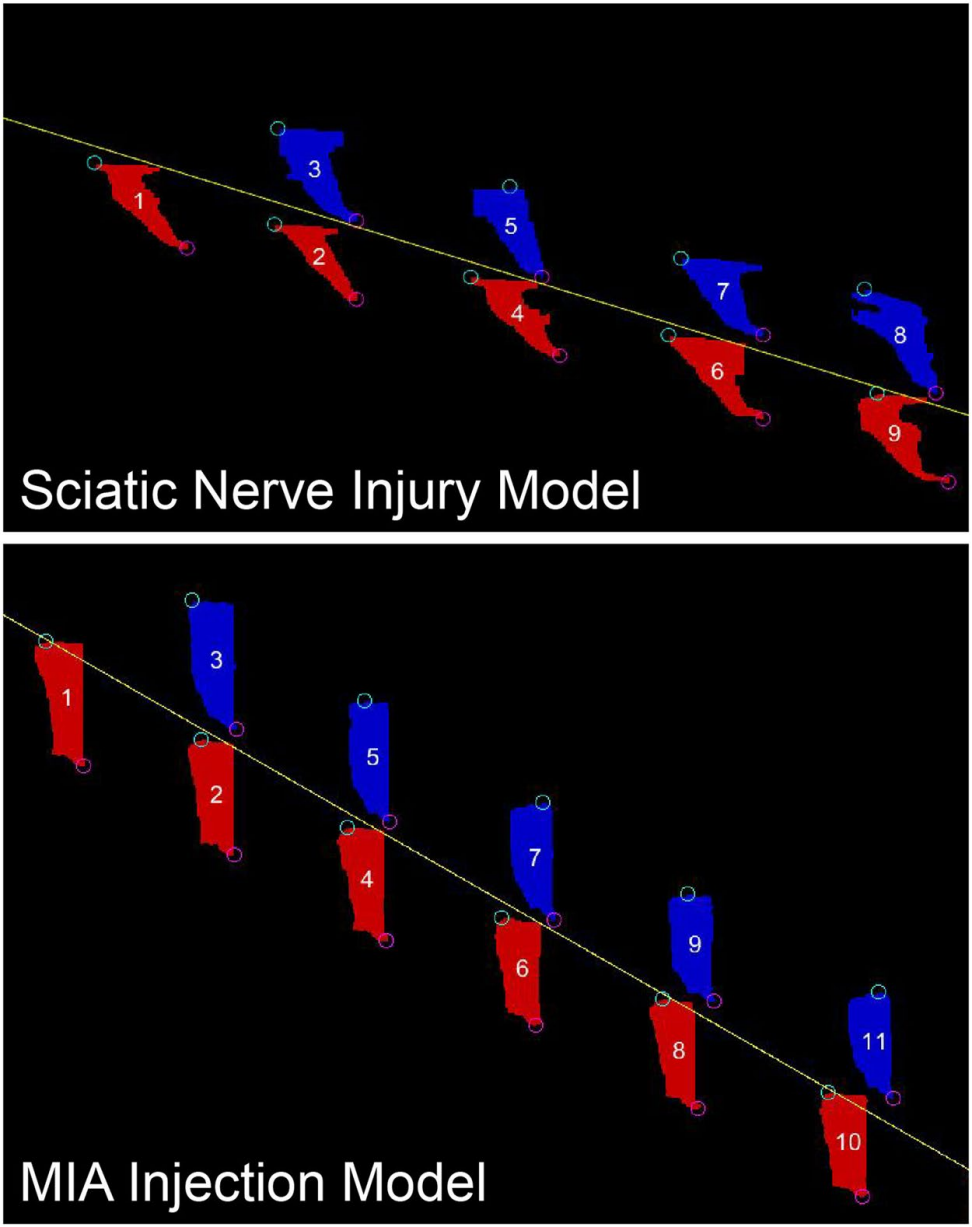
FSTO object distortion is apparent in the sciatic nerve injury versus the MIA injection model. While the GAITOR Suite currently accounts for the timing differences between these models (stance and stride time differences), the FSTO object shape could potentially provide additional insight into model-associated gait changes.

Of further note on the capabilities of the GAITOR Suite, the data presented here include spatiotemporal gait analysis only, and include classic parameters of the quadrupedal gait sequence. Importantly, the open source structure of the GAITOR Suite allows implementation of new or additional measures with relative ease. For example, the sciatic nerve injury model presented in this work can cause the animal’s injured foot to drag or “slip” when walking. This “slip” can be seen clearly in the FSTO images as a consistent artefact (Fig. 12). Since FSTO object data is saved by AGATHAv2, quantifying the foot drag in these animals could be possible by measuring FSTO object distortion. As such, lab groups can tailor GAITOR Suite outputs to best characterize their models. GAITOR Suite software has been provided with the intention of transparency and accessibility, and the code is customizable by individuals with basic MATLAB programming experience. Additionally, the data in this study were collected and processed by both our group and our collaborators, demonstrating the GAITOR Suite’s utility for non-expert system users.

### Elbow Contracture Model

In the elbow contracture model, injured animals developed an antalgic gait compensation evident in both the hind and fore limbs. In the fore limbs, animals had lower left duty factors, indicating less time than expected spent on their injured left fore limbs. In the hind limbs, duty factors were also elevated for the right limb, which is consistent with a gait compensation developed to protect the injured left fore limb. Injured animals were imbalanced in the fore limbs, but not in the hind limbs, indicating a more pronounced alteration of fore limb gait (as would have been expected for a fore limb injury).

The Long-Evans animals used in this study had areas of white and black fur, demonstrating the adaptability of the GAITOR Suite for different colour animals. Additionally, these data demonstrate the GAITOR Suite’s ability to track both fore and hind limb spatiotemporal gait characteristics, a feature previously unavailable in our first version of the AGATHA software^32^. Our group has focused our previous work on assessing the spatiotemporal and dynamic gait characteristics of the hind limbs, as quantitative measures of changes associated with knee osteoarthritis. However, by expanding the functionality of the GAITOR Suite to include fore paw analysis, the suite may be applied to a wider variety of models (such as elbow contracture).

### Spinal Cord Injury Model

In the spinal cord injury model, animals developed a severe antalgic gait in both the fore and hind limbs. In the fore limbs, animals had increased duty factor in the right fore limb, creating an imbalance indicative of a limp. Alternatively, in the hind limbs, animals had increased duty factor in the left hind limb and decreased duty factor in the right hind limb, again creating an imbalance. The opposite findings in the fore and hind limbs highlight the natural trotting gait of rodents, where the right fore limb is in contact with the ground at the same time as the left hind limb. These pairs should experience roughly the same changes in duty factor in order to maintain a synchronized gait pattern, but not all models show *significant* changes in both hind and fore limb gaits. Additionally, animals walked with wider step widths to create a more stable base of support. Changes observed in both hind and fore limb pairs may indicate a more severe compensation, or a physiologic inability to compensate exclusively with fore limbs in a fore limb driven gait alteration.

Again, these data demonstrate the GAITOR Suite’s ability to assess quadrupedal gait compensations. Here, gait compensations in response to injury were not isolated to a single limb pair. No fore limb differences were observed in either the MIA or sciatic nerve transection models, while both fore and hind limb differences were identified in both the elbow contracture and spinal cord injury models.

### Study Constraints

This work does not aim to provide an in-depth analysis of each of the models discussed above. Rather, this work aims to present the potential utility of the GAITOR Suite in several models and demonstrate its potential as an adaptable, open source platform for rodent gait analysis. As such, we acknowledge that these data present only a snap-shot of the gait compensations in the models described. since they represent relatively small cohorts at single time points, there is a possibility of false discoveries. To fully characterize the gait compensations associated with these models, full scale studies assessing individual models (including multiple time points) are necessary. As an example, Lakes and Allen recently published a more complete assessment of the MIA model presented in this work^31^. Thus, while the gait compensations identified in this paper are promising and explainable, they should not be interpreted as comprehensive gait profiles.

Additionally, not all outcomes presented in this work or calculated by the GAITOR Suite are independent variables. When characterizing gait compensations, particularly when attempting to place them in the context of clinically analogous compensations, a single gait variable is not sufficient. While not all outcomes presented are independent variables, they have been included to better characterize gait changes when taken together as a set of descriptive parameters. These outcomes are not presented to over-emphasize the significance of changes in response to disease and injury, but to provide a more comprehensive picture of the observed adaptations than a single measure alone.

### Resources for Using the GAITOR Suite

The GAITOR Suite software has been made available to the research community at our lab websites (www.orthobme.com/resources and www.GAITOR.org). A list of materials and build instructions for EDGAR are also provided, along with recommendations for gait video collection and arena setup, at www.GAITOR.org. The basic GAITOR Suite system (spatiotemporal gait only) can be constructed for under $1,500USD, including the cost of an academic MATLAB license.

The GAITOR Suite was designed with the intention of quick startup in new labs. Basic functionality of the code should be robust and compatible with most EDGAR arena setups without customization. However, since the main AGATHAv2 code saves all necessary gait data, adaptations for individual labs are easily made within the GAITORCalc code, where users can add new calculations or modify calculations to meet laboratory needs. Furthermore, while our software has been written in MATLAB (which does require a purchased license), the open source nature of the code may allow groups to produce adaptations of the GAITOR Suite for use in open source platforms, like GNU Octave”. In addition to the basic GAITOR Suite system, a high-speed camera to collect videos is required. However, the prices for these devices vary widely depending on research group needs (≈$500-$30,000USD), and thus camera cost has not been included in the estimated cost of the system. Nonetheless, current commercial rodent gait analysis systems typically sell in the range of $50,000USD, and achieving a fully functional construction of the spatiotemporal GAITOR Suite system is viable at under 10–20% the cost of commercial systems.

It is also worth noting, the GAITOR Suite includes dynamic data processing tools for groups who wish to include ground reaction force data in their gait analysis. A dynamic gait capable build of EDGAR has been described in our previous work^26^, and full build instructions are available at www.GAITOR.org. However, the necessary hardware to sensitively detect ground reaction force changes in rodents is expensive. As such, these tools have only been used by the developing lab to date, and data on this expansion of the GAITOR Suite are not included in this publication. Nonetheless, this technology has also been open sourced. For many groups, spatiotemporal gait analysis is sufficient to detect gait compensations associated with their models.

### Overall Impact of the GAITOR Suite

Semi-quantitative assessments of pain and disability remain frequently reported in the literature, despite known concerns with the reliability and repeatability of these results^33,34^. Gait analysis provides robust quantitative measures for preclinical models; however, collecting gait data can be expensive, and processing gait data can be tedious. GAITOR Suite hardware (EDGAR) is simple and relatively cheap to construct, and GAITOR Suite software largely automates the most time-consuming components of gait analysis. In this manner, the goal for the GAITOR Suite is to increase access to consistent, quantitative gait assessment for preclinical researchers and increase the robustness of gait characterization in rodent models.
